# Developing Iranian sub-national Primary Health Care Measurement Framework: a study protocol

**DOI:** 10.1017/S1463423622000469

**Published:** 2022-10-11

**Authors:** Ramin Rezapour, Ardeshir Khosravi, Mostafa Farahbakhsh, Elham Ahmadnezhad, Saber Azami-Aghdash, Jafar Sadegh Tabrizi

**Affiliations:** 1 Tabriz Health Services Management Research Center, Tabriz University of Medical Sciences, Tabriz, Iran; 2 Center for Primary Health Care Management, Ministry of Health and Medical Education, Tehran, Iran; 3 Research Center of Psychiatry and Behavioral Sciences, Tabriz University of Medical Sciences, Tabriz, Iran; 4 National Institute of Health Research, Tehran University of Medical Sciences, Tehran, Iran

**Keywords:** framework, Iran, performance measurement, Primary Health Care

## Abstract

**Background:**

Developing an effective system for measurement and improvement of primary health care (PHC) based on the conditions and characteristics of the countries’ health systems is one of the World Health Organization (WHO) recommendations.

**Aims:**

This study will aim to develop a framework to assess the Iranian sub-national PHC system performance using the WHO measurement framework for PHC.

**Methods/designs:**

This is a mix-method study with a triangulation design. The Iranian sub-national PHC Measurement Framework (PHCMF) will be developed through a review of the WHO’s PHC measurement conceptual framework (for selecting key performance indicators (KPIs)), literature review (academic database), PHC-related national documents, consultations with national experts, and the Delphi technique for finalizing the framework. The required data for calculating selected KPIs is expected to encompass qualitative and quantitative data. *Discussion:* Iranian PHC system performance is not measured based on the holistic and scientific framework and international standards. The information obtained from this project will guide managers and policymakers to be aware of the current situation and the success rate of the PHC system in achieving the desired goals, as well as identify strengths and weaknesses of the PHC system and provide the solution to better policy formulation.

## Background

Despite widespread agreement on the success of Primary Health Care (PHC) in saving and improving lives, half of the world’s population still does not have access to essential health care (WHO, 2017). Every year, 8.6 million people die from diseases that can be prevented and treated by PHC (Kruk *et al.*, 2018). In recent decades, the focus on PHC strengthening and improving PHC performance were increased, especially in developing countries (Baltussen *et al.*, 2002). Assessment and measurements of PHC performance play an increasingly influential role in health system reform (Rezapour *et al.*, 2019). Stakeholders need this information to lead decisions toward better outcomes (Smith *et al.*, 2009). In the past, decisions and planning have not always been evidence-based, and performance improvement programs were carried out regardless of the correct evaluation outcome and often with political arguments or the interests of specific groups (Moosavi *et al.,* 2020). Governments must take responsibility for their actions, requiring evidence and accurate scientific performance assessment (Derakhshani *et al*., 2021). Accordingly, World Health Organization (WHO), in cooperation with other international organizations, including the United Nations Children’s Fund (UNICEF), the World Bank (WB), the Bill & Melinda Gates Foundation, scientific and academic groups, and departments, launched a Primary Health Care Performance Initiative (PHCPI) to create a special contribution to transforming the global state of PHC (Veillard *et al.*, 2017). PHCPI has been developed to reduce and eliminate the critical gap in measuring and improving PHCs (PHCPI, 2018b). The health system revisions have been reconsidered in recent years based on the new PHC. In this regard, on the 40th anniversary of the Alma-Ata Declaration, the Eastern Mediterranean Regional Office (EMRO) introduced the Primary Health Care Measurement and Improvement Initiative (PHCMI) as a regional plan for implementation in member states (Veillard *et al.*, 2017). The PHCMI’s overall goal is to provide a set of tools (WHO, 2022). Member states can integrate these tools into their current PHC assessment programs and use them to identify deficiencies and challenges of the service delivery system. Performance information can lead to appropriate policymaking and regulation (Barzegar and Djazayery, 1981; Shadpour, 2000).

Iran participated in the initiative introductory workshop with other EMRO member states like Afghanistan, Bahrain, Iraq, Lebanon, Oman, United Arab Emirate, and others. Accordingly, since 2019, Iran has started the PHCMI program seriously to improve PHC. The experience of national implementation of the PHCMI in cooperation with WHO has highlighted the strengths and weaknesses of Iran’s PHC.

PHC in Iran is provided at three levels: national, provincial, and district (Appendix 1). Provincial and district levels are known as sub-national levels in this study. There are 31 provinces and 464 districts in Iran’s health system. There are provincial health centres and district health networks at the province and district levels. District health networks are organized similarly throughout all provinces (Tabrizi *et al.*, 2022).

Around 35 years ago, the Iranian PHC network was developed across the country. Each village or group of villages in rural areas has its own Health House (HH), which is staffed by qualified community health workers known as “Behvarz”. In rural areas, there are also Rural Comprehensive Health Centers (RCHCs) that serve about five HHs. A physician (GP), numerous health technicians, and a general administrator run the RCHC. Similar-sized Health Posts (HP) and Urban Comprehensive Health Centers (UCHC) have been established in urban regions. HPs are staffed by “Moragheb-e-Salamat” community health workers and health volunteer women from the same community, while UCHCs are staffed by a physician and health technicians) and provide public health services comparable to those provided by RCHCs. District Health Centers are responsible for supervising and administering the entire network. They are primarily responsible for managing and coordinating the activities of rural and urban health centres, collaborating with more specialised district hospitals and other public medical or paramedical establishments (Tabrizi *et al.*, 2022).

Key performance indicators (KPIs) provided in the PHCMI project were suitable for the national level. Because the second level of PHC differs from the third level and the measures and interventions that are carried out at this level are unique, it is necessary to adjust the setting of national KPIs to the province and district as a sub-national level. Developing a provincial and district framework for PHC measurement will provide necessary information to compare the performance of different provinces and districts and develop improvement plans according to the specific conditions and characteristics of the provinces and districts.

Following the participation of Iran delegates in the first regional consultative meeting PHC for Universal Health Coverage from 29 July to 1 August 2019, regarding the formal start of the PHCMI, an internal meeting was organized with the participation of the National Institution of Health Research (NIHR) and WHO CO focal persons to finalize the PHCMI committee. The core team members consist of the director general for PHC at the Ministry of Health and Medical Education (MOHME), the head of statistics and health indicators at MOHME, experts in the field of health management, health economics and epidemiology at NIHR, experts in the departments of communicable and non-communicable diseases, family health, and environmental health at MOHME.

**Figure 1.**
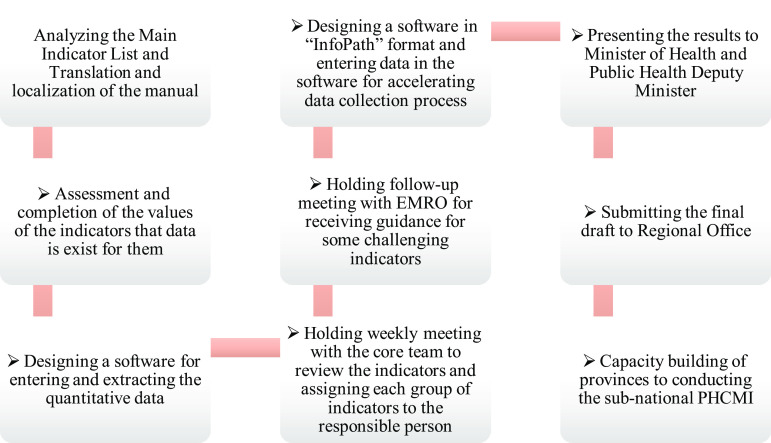
Actions that are taken in the national phase of the PHCMI project

After designating the core team, the internal meetings were coordinated weekly and the nine actions were taken (Figure 1).

This study will aim to develop and implement the Iranian sub-national PHCMF. Besides that, we will identify the weaknesses, conduct a gap analysis based on the results, and develop an improvement plan based on evidence gathered during the implementation of the framework. The necessary data will be collected from health houses, health posts, and health centers. The implementation of this framework assists local managers and policymakers in formulating better policies and strategies based on available data.

## Primary health care measurement conceptual framework

**Figure 2.**
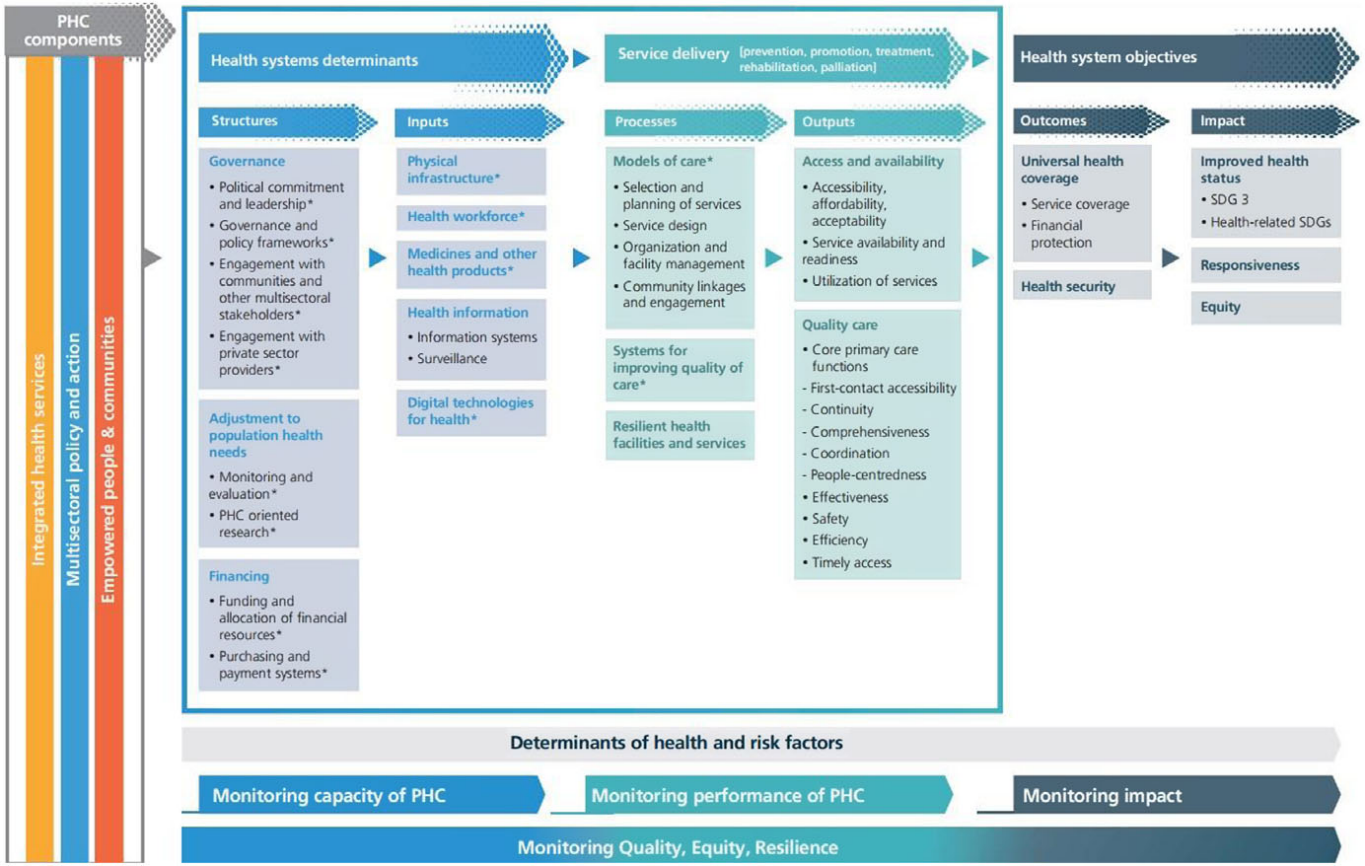
PHC conceptual framework Source: WHO

The PHCM framework was provided by WHO to continuously strengthen PHC and support member states to assess, track, and monitor PHC performance improvement. This framework was developed based on and supported the levers of the operational framework introduced by PHCPI. The PHCM framework was organized in three ways, by results chain domain: structures, inputs, processes, outputs, outcomes, and impact; by PHC domain to support PHC orientation of health systems; and by PHC monitoring dimensions (WHO, 2022) (Figure 2).

About 121 KPIs (2018) and 89 KPIs (2022) were introduced by WHO to assess each of these framework levers (WHO, 2018b; 2022). These KPIs and frameworks have been developed based on the technical review, consultation with countries, and PHC academics and experts.

**Figure 3.**
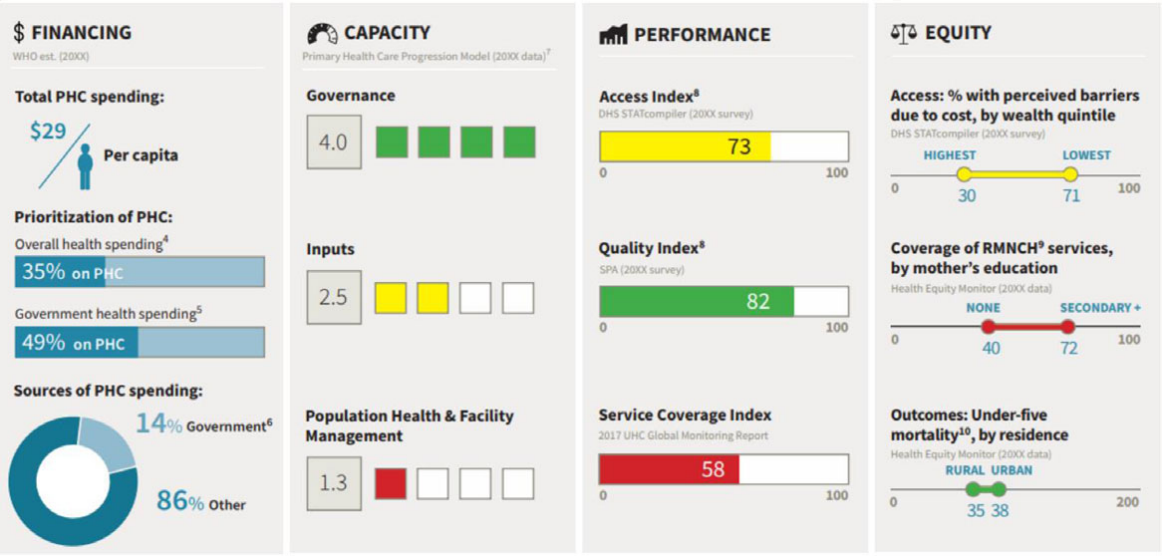
PHC vital sign profile Source: PHCPI

The need for criteria to provide a summary of PHC performance, as well as detailed information on PHC performance, led to the provide a core set of KPIs in the form of PHC vital Signs profile (VSP)(PHCPI, 2021). PHC vital signs profile (VSP) is a core set of 26 KPIs. This collection, obtained from 121 KPIs, provides a snapshot of the most important aspects of the PHC performance and allows countries to compare their performance (Figure 3).

The VSP is categorized into four pillars: financing, capacity, performance, and equity. Each pillar of VSP provides essential information for understanding the functioning of the PHC system. However, all pillars cannot be easily measured through the available quantitative indicators. In particular, the capacity pillar is measured very poorly by existing quantitative indicators. Due to the lack of strong quantitative indicators to assess the capacity pillar, PHCPI has introduced a progression model, a new measurement tool using combined methods. The progression model includes 33 measures (PHCPI 2018a; 2021).

## Method

This is a mix-method study with a triangulation design (Creswell and Clark, 2017). In this method, qualitative and quantitative methods start parallel. We will use the mixed methodology since we needed to extract the indicators and select them through an experts view (Qualitative study), we needed to measure the quantitative indicators in the facility (Quantitative study), and we needed to develop and validate the framework through an experts view (Qualitative study). Reviewing the indicators of the WHO and using the experiences of national and sub-national experts in the development and implementation of the framework as well as the pilot test of the framework in the two provinces will strengthen the study.

**Figure 4.**
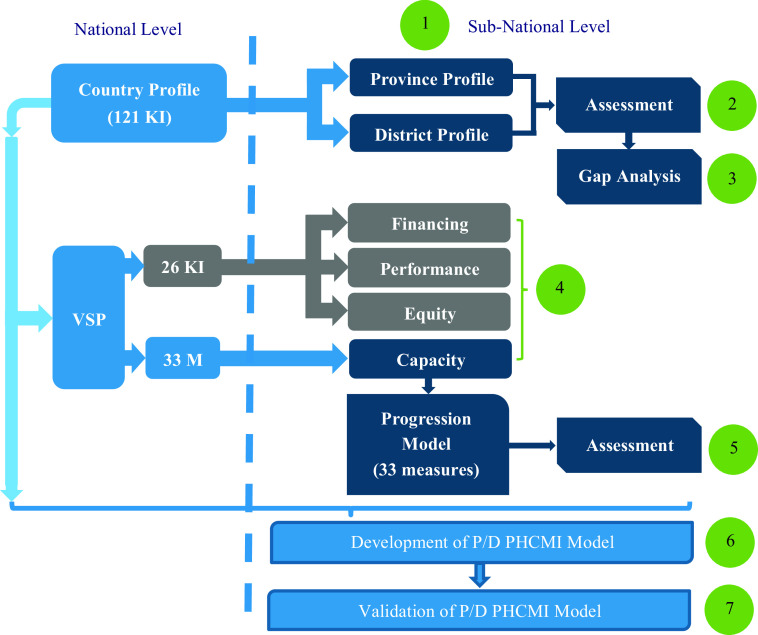
Iranian sub-national primary health care measurement and improvement model development process (numbers in green)

This study will conduct in seven steps (Figure 4). WHO released a report entitled” Primary health care measurement framework and indicators: monitoring health systems through a primary health care lens” in 2022 that suggests six specific steps for implementing the PHC monitoring framework at the subnational level (WHO, 2022). Aligning the general policies of the country’s health system with the global framework, selecting indicators in accordance with the country’s strategies and programs, identifying gaps in the health system, determining the baseline value and target for indicators, and identifying valid data sources are among the recommendations of WHO. The study steps are designed based on the steps proposed by the WHO.

Step 1: Determining the key performance indicators (KPIs) according to the Iranian sub-national PHC system capacities

The first step of the study will be conducted in three parts, including a literature review, an expert panel meeting, and the Delphi survey. More detailed explanations about those parts are reported below.

## Literature review

A systematic search will be conducted using Scopus and PubMed databases to identify studies related to KPIs. The initial search keywords will be the following: performance assessment, performance measurement, performance indicator, and PHC. Studies focusing on specific populations such as the elderly and children or diseases such as diabetes and hypertension will be excluded. The research team will review selected KPIs and prepare a preliminary list of suitable KPIs.

## Expert panel

The expert panel will review KPIs introduced by WHO and KPIs extracted from the literature review to prepare an initial list of KPIs suitable for the sub-national level. We will consider inviting experts with at least 5 years of professional experience in the PHC, and being responsible as an executive director in the PHC, at least at the district level and above. Four basic criteria will be used to review KPIs, including measurability, importance, relevance, and actionability (Table 1).

**Table 1.** Key performance indicator (KPI) selection criteria

Criteria	Description
Feasibility	There is the necessary infrastructure and conditions to measure the KPIs
Importance	The KPIs reflect key characteristics of the primary health care (PHC) performance
Relevance	The indicator is related to the main processes of PHC
Actionability	Actionability

The PHCM framework comprises several levers such as governance and policy, funding and allocation of resources, digital technologies for health, systems for improving the quality of care, etc. (WHO, 2022). Expert members will be selected according to the different levers. We plan to purposively select 20 experts in PHC performance measurement (including experts from the public health deputy of MOHME and PHC experts from the sub-national level in the field of health economics and health system financing, quality assessment, health system management, and health systems performance assessment). If we do not reach data saturation with these 20 experts, we will continue to add experts until the data saturation is reached.

After reviewing the KPIs and selecting suitable KPIs, alternative KPIs will be suggested by experts. In order to introduce alternative KPIs, extracted KPIs from a literature review and the list of Iranian PHC quality assessment indicators, national documents related to PHC, and other related documents will be reviewed by an expert panel. Then a list of KPIs for assessing PHC at the sub-national level will be prepared.

## Delphi survey

We will conduct a two-round classic Delphi survey (Ha and M, 2002) with key experts involved in PHC performance measurement at the national and sub-national levels. The Delphi survey will be used to reach a consensus on the KPIs. At least 20 relevant experts will perform Delphi survey. Criteria for selecting KPIs according to the criteria introduced by the WHO will include: importance, measurability, and relevance. KPIs with a median of less than 4 will be excluded from the study.

### Data analysis

KPIs with a median score of >4 and <7 will be entered into the second round. Also, KPIs with a median score higher than seven will be accepted as final KPIs. After selecting and finalizing the KPIs, metadata will be developed for each of the KPIs (Appendix 2).

Step 2. Measuring the Iranian sub-national PHC based on the determined KPIs

The selected KPIs in the previous step based on data collection sources will be classified into three groups.

- Based on the PHC routine information systems;
- Based on the facility surveys;
- Based on the population (household) surveys

For collecting the KPIs required data, two provinces will be selected as a pilot to identify the challenges in data collection and availability and system capacity to apply the framework across the country. The following steps will be done at the sub-national level:

1. Participating in the workshops and learning sessions to learn about the PHCM framework and KPIs
2. Establishing a PHCM framework workgroup at the sub-national level
3. Planning human resources for the program at the sub-national level
4. Advertising, promotion, and attracting stakeholders’ support
5. Holding training sessions
6. Preparing for data collection
7. Fieldwork and data collection
8. Data entry and data management
9. Program budget and costs
10. Program timeline and schedule

A: There are four PHC routine information databases, including Integrated Health System (IHS) entitled “SIB” Health Information Software (HIS) entitled “NAB” PARSA system, and the integrated information system entitled “SINA”. All information about households and the type of services provided in UCHCs, RCHCs, HHs, and HPs are recorded in these systems. Mortality indicators will be extracted by experienced and trained staff from the death registration systems and the National Registration Organization (NRO).

Sampling: We will use the census to measure these KPIs and select all PHC facilities at the sub-national level.

Data analysis: The KPIs will be calculated based on metadata (Appendix 2). The metadata includes KPIs rationality, level, formula, target, frequency of measurement, etc. We will use Excel software to calculate KPIs based on the formula.

B: Health Facility Survey: Service delivery assessment indicators and availability of equipment and instructions indicators

Sampling: The statistical population will include all active PHC facilities at the subnational level. CHCs, RHCs, and attached facilities will be considered as sampling units. A random sampling method will be used. The following formula will be used to calculate the sample size.

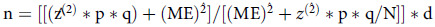

(n = sample size, z = 95% confidence level (1.96), p = Ratio of the desired predicted attribute of the service delivery unit, q = 1−p, N = number of facilities, d = design effect)

Data collection: We will use existing standard tools to assess KPIs. Existing standard questionnaires and checklists will be used to collect the data. Observation of service delivery process and equipment, document review, and interview with the provider will be used for data collection.

Data analysis: We will use descriptive statistics, including frequency, relative frequency, mean and standard deviation (SD), to describe the KPIs. SPSS (Statistical Package for Social Science) software version 16 will be used for data analysis.

C: Household Survey: Demographic and Health Surveys (DHS), Responsiveness Assessment Indicators, and Multiple Indicator Cluster Surveys (MICS)

This group’s KPIs will be gathered and analyzed using a methodology similar to group B.

Step 3. Gaps analysis of the Iranian Sub National PHC system based on the KPIs results

This step will evaluate the degree to which PHC performance is currently aligned with the ideal and anticipated state. A gap analysis will be performed in the following three sections:

First, compare KPI performance to target and expectations: The value of the KPIs will be compared with the predetermined target value (optimal/expected) to identify the gap. Positive scores will show that PHC performance is better than anticipated, while negative scores will show a performance gap. A zero score will imply that there is no gap and that the performance is as predicted.

Second, expert panel meetings with key stakeholders: The reasons for the gap and solutions to address it will be reviewed by experts. Each KPI will be reviewed by relevant stakeholders (for example, infectious disease death rates by Non-Communicable Disease (NCD) department experts).

Third, review and classify the gap analysis results: Finally, causes and solutions will be reviewed and categorized based on KPIs priorities.

Step 4. Adjusting the progression model according to the Iranian sub-national PHC system capacities

PHC progression model is a mixed-methods assessment tool for measuring the foundational capacities of PHC. The model consists of 32 measures. To adjust and localize the measures with the sub-national capacity, 20 experts will review the 33 measures of the progression model in the PHC area through an online questionnaire. Participants will score each of the measures on a 4-point Likert scale based on the four criteria listed in Table 1. They will also be invited to suggest additional measures. The results’ analysis and summarization will follow the first step’s analysis method.

Step 5. Implementation of the progression model at the Iranian sub-national PHC system

We will implement the progression model in three steps according to the WHO protocol (PHCPI, 2019):

#### Step one: planning

Understanding the progression model and how to use it will be the goal of this step. The main elements that will influence how well the assessment process proceeds will also be identified. The following activities will be done: (1) Defining assessment parameters, (2) Forming an assessment team and defining the roles and responsibilities of each member, (3) reviewing the progression model and defining the key terms, (4) Identifying data sources, (5) Setting a data collection plan (Translating general data collection plans into detailed and operational plans: including a decision on sampling, assignment of tasks and responsibilities, and determination of time frame and budget).

#### Step two: assess

This step will aim to complete the data collection and internal assessment program. The actions that will be conducted at this step include (6) Data collection (Collecting all required data, including quantitative and qualitative data and documents), (7) Data analysis (Extracting relevant evidence, summarizing, and organizing information based on measures), (8) Conducting internal assessment (Using all the evidence to complete an internal scoring and assign a score between 1 and 4 to each measure).

#### Step three: finalize

This step will be performed to conduct an external review to ensure the results of the progression model and verification of existing findings, and finalization of scores. Also, the results of the progression model will be integrated into the VSP. At this step, three actions will be done, including (9) Conducting external validation (To ensure that the findings are rooted in the available evidence. Several measures will be randomly selected for verification. The external validation method will be the same as the internal assessment following the WHO guidelines (PHCPI, 2019). If the external validation results differ from the internal assessment, all external validation criteria will be re-examined to ensure that the results are correct), (10) Resolving discrepancies (Resolving any differences between internal and external scores and determining the final score for each of the 33 measures), (11) Approving final scores and placement of scores in the VSP.

Step 6. Designing of the Iranian sub-national PHCM framework

Using the results of the previous five steps, the initial Iranian sub-national PHCM framework will be developed. To develop a framework, the research team and three experts outside the team will work together. The expert’s selection process will be similar to the first step. The WHO framework will be considered a basis of the Iranian sub-national PHCM framework (WHO, 2022). The KPIs that will collect successfully will be categorized into levers (see Figure 1).

Step 7. Validation of the Iranian sub-national PHCM framework

After determining the initial framework, the Delphi survey will be used to determine the validity of the framework. Accordingly, the initial framework with a detailed description of levers and KPIs will be sent to 20 experts throughout the Delphi questionnaire. To assess the validity of the framework, we will consider 12 items, including (1) Applicability of the framework, (2) Adaptation of the proposed framework to the upstream documents, (3) Ability to accept the framework by stakeholders, (4) Efficiency, (5) Flexibility, (6) Effectiveness, (7) Simplicity, (8) Coherence and integration between framework components, (9) Comprehensiveness, (10) Sequence of framework components (11) Proportion and balance between framework components, and (12) Overall.

Experts will score each criterion on a 4-point Likert scale (completely agree to disagree). The framework’s validity will be assessed using a modified content validity index and kappa. Kappa above 0.40 will be considered (fair), between (.60) and 0.74 (good) and above 0.74 (Excellent).

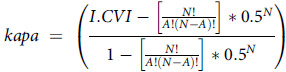




*N* = number of experts; *A* = number of experts with completely agree score

## Discussion

Experts from many international organizations have visited and appreciated PHC’s performance in Iran because of its reputation internationally (Gharaee *et al.*, 2019). Many achievements and milestones have been attained in promoting health indices after approximately 35 years of PHC initiative in Iran in the form of health care networks (Lankarani *et al.*, 2013). The development of health networks occurred in three phases, including developing the “Behvarz” program, developing a family practice program in rural areas and urban communities, and making progress towards developing family practice in suburban areas and cities with a population of more than 20 000 people (WHO, 2018a). Since the “Behvarz” program’s commencement, the population’s life expectancy has grown, rising from 55.7 years in 1976 to 75.5 years in 2015 (WHO, 2015). The PHC system played a significant role in this outcome. With the use of a significant number of service providers in the family practice programs, health services are now more widely available in urban areas, and people are more motivated to use services related to nutrition, mental health, and NCDs (WHO, 2018a).

Despite the tremendous success of the Iranian PHC system in addressing the basic needs of the people, most of the frameworks and models developed to measure the Iranian health system’s performance have focused more on clinical and hospital services (Fadaizadeh *et al.*, 2012; Lotfi *et al.*, 2015; Bahadori *et al.*, 2016) and PHC performance measurement has rarely been studied. Few studies on PHC performance have been conducted, such as the providing PHC quality assessment framework (Rezapour *et al.*, 2019), providing a conceptual framework for public health performance based on health determinants (Jahanmehr *et al.*, 2015), and providing performance indicators at the city level (Tabrizi *et al.*, 2018). The development of the Iranian sub-national PHCM framework, based on a comprehensive global framework and including all dimensions and levers of the PHC system from structure to impact, is a novel and essential topic. Therefore, in this study, we want to develop this framework.

The WHO Regional Office for the Eastern Mediterranean (EMRO) has suggested that member states adjust the PHCM framework based on local and national conditions. Since the PHC system in Iran is structured differently from those in other countries, and the levels of care within this structure are also different from one another, the development and implementation of the Iranian PHCM framework will be on the agenda.

According to member states’ experiences and perspectives, in cooperation with PHCPI groups and other organizations, the WHO developed the PHC measuring framework and PHC progression model. The developed framework was piloted in five countries for refinement, completion, and finalization before being updated and distributed to the member states for implementation. This study will develop the Iranian sub-national PHCM framework using the PHCMI and progression model development methodology, global experiences, and national experts’ meetings. The framework will be piloted in the two selected provinces and districts to refine, complete, and finalize.

Improvement of quality, patient safety, and customer satisfaction is achievable by implementing the Iranian sub-national PHCM framework. It is anticipated that the current study will provide a suitable and valuable framework for assessing and improving PHC performance at the sub-national level.

## Strengths and limitations

According to our best knowledge and literature review results, this study will provide a scientific and evidence-based tool to measure the Iranian sub-national PHC. Based on the Iranian Sub National PHCM framework, we address possible interventions for the weaknesses and challenges. We will provide a tool used by policymakers to compare the performance of different provinces and districts. Also, we will use multi-sectorial team-based approaches to maximize national and subnational and academic and executive staff cooperation.

However, the present study will have two major limitations. The first limitation will be the lack of access to private sector data. To overcome this limitation, we will try to engage representatives of the private sector in the project if necessary, and we will also consider incentives for ease of data access. The second limitation will be the lack of access to data of some KPIs due to these confidentialities. We will try to offer the appropriate guarantees required to prevent data from being disclosed to the public in order to access confidential data and keep data security.

The Iranian sub-national PHCM framework, as a comprehensive and scientific tool, will play a vital role in translating goals into action plans and continuous quality improvement in the PHC system. Therefore, it is recommended to use this framework and suggested KPIs for quantitative and qualitative assessment of the Iranian PHC system performance, including the resources and infrastructure required to provide services, human resource management, quality of care, safety, and meeting the population health needs and also provision of universal health coverage.

## Data Availability

Not applicable in this section.

## Supplementary material

For supplementary material accompanying this paper visit http://doi.org/10.1017/S1463423622000469. click here to view supplementary material
