# Supplementary material for: Developing Iranian sub-national Primary Health Care Measurement Framework: a study protocol
Source: Prim Health Care Res Dev. 2022 Oct 11;23:e62. doi: 10.1017/S1463423622000469 (PMC9641646; doi:10.1017/S1463423622000469)
Supplement: Supplementary file 1 [file phcsup.zip › S1463423622000469sup001.docx]

| **A: Domain** |
| --- |
| **Indicator Name** |
| **Rational** |
| **Department** |
| **Indicator Level** |
| **Numerator** |
| **Data Source** |
| **Denominator statement** |
| **Target** |
| **Data reported as** |
| **Frequency of Measurement** |
| **Formula** |
| **References** |

based on the classification provided by Donabedian: structural, process and outcome

Indicator report format; For example: percentage or ratio

ideal value as expected from the indicator

The time period which the indicator is reported, for example: monthly or annually

Articles, reports and other sources of information used to extract the indicator

How to calculate the indicator, which is presented in the form of a fraction, including Numerator and denominator

The source or location where the indicator data will be collected

A brief and concise definition of the indicator and the importance and necessity of its use

**Iranian PHCMI Indicators Identity**

The indicators domain will be determined according to the conceptual framework

The unit or department that is primarily responsible for collecting data
