# Supplementary material for: Developing Iranian sub-national Primary Health Care Measurement Framework: a study protocol
Source: Prim Health Care Res Dev. 2022 Oct 11;23:e62. doi: 10.1017/S1463423622000469 (PMC9641646; doi:10.1017/S1463423622000469)
Supplement: Supplementary file 1 [file phcsup.zip › S1463423622000469sup002.docx]

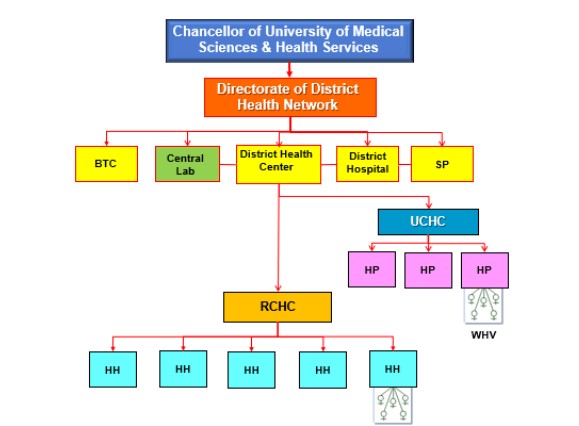


*Additional File 1. The structure of primary health care in Iran*

| Number of Facilities | |
| --- | --- |
| Province University of Medical Sciences | 31 |
| Vice Chancellor for Healthcare (PHC) | 61 |
| District health center Network | 464 |
| Urban Health Center | 3236 |
| Rural Health Center | 2929 |
| Health Post | 5640 |
| Health House | 17958 |
